# Leveraging intermediaries’ skillsets to build implementation research and practice infrastructure: a qualitative case study

**DOI:** 10.1186/s43058-025-00765-2

**Published:** 2025-08-02

**Authors:** Stephanie P. Brooks, Cody Alba, Stephanie Montesanti, Denise Thomson, Sara N. Davison, Kathryn Graham, Kate Storey

**Affiliations:** 1 Learning Health System Team, Alberta SPOR SUPPORT Unit, Department of Medicine, University of Alberta, Edmonton Clinic Health Academy, 11405 87 Ave NW, Edmonton, AB T6G 1C9 Canada; 2 School of Public Health, University of Alberta, 3-300 Edmonton Clinic Health Academy, 11405–87 Ave NW, Edmonton, Alberta, T6G 1C9 Canada; 3 Department of Medicine, University of Alberta, 8-105 Clinical Sciences Building, 11350 83 Ave NW, Edmonton, Alberta, T6G 2G Canada; 4 Community of Health Sciences, University of Calgary, Cal Wenzel Precision Health Building, 3280 Hospital Dr NW, Calgary, AB T2N 4Z6 Canada; 5 Faculty of Rehabilitation Medicine, University of Alberta, Corbett Hall, 8295 114 St NW, Edmonton, AB T6G 2G4 Canada

**Keywords:** Implementation Science, Learning Health Systems, Co-Design, Facilitation, Intermediaries, Partnership

## Abstract

**Background:**

Implementation science as a field is rapidly advancing. Moreover, implementation science plays a pivotal role in driving learning health systems to better realize health outcomes and impact for our communities. Yet, few reports detail the infrastructures that underpin embedding and managing implementation science activities. Furthermore, there is little guidance for designing these infrastructures (people-powered and/or inanimate supports essential for embedding implementation research questions in pilot, spread and scale initiatives) to address local research and practice needs. The Implementation Science Collaborative is one such infrastructure in Alberta, Canada that leverages existing expertise in implementation research and practice to facilitate embedded implementation research and increase the success rates of health innovation implementation for better health outcomes. This study sought to provide actionable recommendations for designing effective implementation infrastructure by examining the co-design of the Implementation Science Collaborative.

**Methods:**

We conducted a longitudinal case study (2018–2021) of the Implementation Science Collaborative using document analysis and semi-structured interviews. We collected data from initiative planning and operations documents (*n* = 190) and semi-structured interviews with Implementation Science Collaborative members (*n* = 6). We applied the Large-Scale Change Driver Model as the analytical framework for qualitative analysis to generate insights into designing cross-sectoral implementation science infrastructure.

**Results:**

Our analysis showed that infrastructure design and operationalization followed established principles of implementation planning and execution. Implementation intermediaries proved to be effective facilitators as they had the backgrounds required to guide co-design and implementation planning. Their political neutrality in the resulting infrastructure enabled them to address power imbalances among co-design partners. However, strong management leadership remained irreplaceable. Cross-sectoral leadership was essential in fostering and solidifying the partnerships required for supporting the local learning health system.

**Conclusion:**

The study findings highlight the effectiveness of a co-design approach, facilitated by intermediaries, in developing local implementation science infrastructure and management systems as a promising practice to implement for achieving outcomes. This approach enabled the creation of infrastructure designs that meet diverse user needs. However, co-design is a complex process that benefits from both intermediaries’ skills and cross-sectoral leadership knowledge of the local learning health system.

**Supplementary Information:**

The online version contains supplementary material available at 10.1186/s43058-025-00765-2.

Contributions to the literature

- This article contributes to the under-studied area of evidence-based management of implementation infrastructure co-design.
- Highlighting the dynamics of effective leadership and facilitator characteristics allows researchers to study the interactions between the two and inform the best management model for implementation infrastructure co-design.
- Through our research, we also shed light on how to build fit-for-purpose infrastructure that services both implementation researchers and practitioners.

## Background

Both implementation research and practice contribute to advancing the broader field of implementation science [1–3], yet few reports detail the infrastructures that enable teams to study or execute implementation in a sustained way. Implementation research infrastructures include a broad range of people-powered and/or inanimate supports essential for embedding implementation research questions in pilot, spread and scale initiatives (e.g., funding opportunities, research-capacity building programs, and research prioritization mechanisms) [4–6]. The knowledge created through implementation research strengthens implementation practices, thereby increasing the likelihood of creating and sustaining change [7]. Similarly, implementation practice infrastructure, such as implementation support offices, data capture and monitoring systems, accountability policies, and funding cycles enable implementation, scale, spread and sustainment [8]. Ideally, these infrastructures would also empower teams to apply the principles of implementation science into their practice.

Introducing new infrastructure requires substantial investment by organizations in the form of financial and human capital. This investment is required to develop the infrastructure and manage the change associated with its implementation. To address the financial, social, and environmental challenges that arise from infrastructure development, teams across various sectoral and policy settings have begun advocating for the use of principles from implementation science and integrated knowledge translation to plan and execute infrastructure initiatives [9, 10]. In support of these calls to action, previous research has demonstrated the added value of engagement and co-creation that involves all parties interested in or impacted by infrastructure development [9–13].

Co-design (also known as co-creation and co-production) is the process of bringing together transdisciplinary expertise and perspectives of people interested in and/or impacted by a proposed change to build knowledge and meet specific community needs [14, 15]. Authors argue that a co-creation approach for infrastructure development generates organizational and user buy-in, making it implementable [10]. Through systematic reviews and practice reflections, principles and process models have emerged to guide co-design in research generally [16], industry settings [10], and healthcare [13]. However, few studies report the process of co-designing implementation-specific infrastructure that can be institutionalized and managed. Furthermore, guidance is limited on how to operationalize management systems for institutionalizing or sustaining implementation infrastructure. Despite the central role of infrastructure in advancing implementation science and making its principles accessible to implementation practitioners, guidance on designing these infrastructures to meet local research and practice needs is lacking.

This study outlines a co-design process used to support implementation research and practice at the provincial level in Alberta, Canada. It aimed to 1) explore the co-design and management processes underlying an implementation infrastructure implemented at scale, and 2) identify the key players involved. In presenting our examination, we argue that intermediaries serve as effective facilitators because of their training and expertise in co-design and implementation planning. Furthermore, when intermediaries are free from political interests in the resulting infrastructure, they are better positioned to address power imbalances among cross-sectoral actors involved in the co-design activities.

### Context

At the time of this research, Alberta’s healthcare delivery was managed by a single, province-wide health organization, Alberta Health Services (AHS). AHS had invested heavily in transforming into a learning health system [17] to accelerate efforts to a) improve health outcomes, b) improve patient and provider experiences with care, c) ensure safety and job satisfaction for providers, d) increase quality and value of care, and e) work towards equity in healthcare. These are common goals of healthcare organizations known as the quintuple aim [18]. AHS established its learning health system primarily by implementing an Epic electronic health record system [19, 20] and establishing Strategic Clinical Networks™ [21]. Strategic Clinical Networks were teams of research and practice partners mandated to generate knowledge and implement improvements for priority issues in various sectors, populations, and conditions [22]. AHS had worked to establish many important learning health system components; however, implementation science expertise within the organization was fragmented and underpowered to optimize learning health system processes [23, 24].

While AHS was strengthening its learning health system, Canada’s federal health funding agency, the Canadian Institutes for Health Research, began prioritizing support for learning health systems [25] and implementation science [26] to enhance the effective movement of evidence into practice nationwide. The learning health system mandate primarily fell to the provincial Strategy for Patient-Oriented Research (SPOR) Support for People and Patient-Oriented Research and Trials (SUPPORT) Units. These units were established as a platform to support the creation and mobilization of evidence that upheld patient health priorities and engagement. Given the Alberta SPOR SUPPORT Unit (AbSPORU) knowledge mobilization mandate, there was a natural fit for AbSPORU to support AHS in building the implementation research and practice components of their learning health system for advancing the health system towards its quintuple aim goals [23, 24, 27].

In 2018, prominent Canadian implementation scientists identified Alberta as a strong context for generating valuable implementation knowledge. Given the provincial-level health services delivery and data infrastructure, Alberta had the potential to power large-scale multi-site implementation trials to generate knowledge to optimize implementation approaches for different geographies (i.e., urban, rural, remote), populations and sectors. Implementation science champions from the University of Alberta, University of Calgary, AHS, and AbSPORU partnered to organize two province-wide events to explore the need and feasibility of creating infrastructure and subsequently embarked on an implementation infrastructure-building process.

Numerous teams have noted persistent gaps in implementation capacity, infrastructure, and practical guidance for supporting large-scale change in complex health systems [27–29]. These gaps reflect the core challenges in the Large-scale Change Driver Model, which identified leadership, enabling infrastructure, and capacity-building as essential conditions for transformational change [27]. This study reports on the co-design process for designing and operationalizing infrastructure for implementation capacity-building and research to meet the needs of Alberta’s health implementation community. This infrastructure, called the Implementation Science Collaborative, was designed to help accelerate and enable the integration of implementation science teams into the healthcare system by supporting rigorous planning, evaluation, and study of implementation scale, spread, and sustainment initiatives. By documenting the co-design of the Implementation Science Collaborative, this study contributes to emerging theory on the evidence-based management of implementation infrastructure, offering insights into how leadership dynamics, facilitator competencies, and participatory co-design processes can be aligned to build fit-for-purpose infrastructure that serves both implementation researchers and practitioners. A full description of the infrastructure model was published by Flynn et al. [23] and an account of the scope of the Implementation Science Collaborative operations was published by Brooks et al. [24].

An important contextual factor to note is that AbSPORU hosted two events that delved into implementation support needs in the province; from the feedback in these events, a working group developed the concept of the Implementation Science Collaborative. Discussions about leadership of the Collaborative, and the appropriate home for it in the provincial health research ecosystem, eventually led to AbSPORU formally taking on the role as the Implementation Science Collaborative host. In 2020, after two years of foundational design work, AbSPORU formally accepted leadership responsibilities for the Implementation Science Collaborative. One of the strengths of AbSPORU as the co-design host was the cross-organizational leadership model for the team of intermediaries who facilitated the infrastructure co-design. These intermediaries, known as Implementation Science Collaborative implementation facilitators, brought expertise in implementation science, knowledge translation, and applied research, alongside skills in relationship-building, co-design, group facilitation, and systems thinking. They also had experience engaging interest-holders across sectors and navigating organizational dynamics—skills increasingly recognized as foundational to building implementation capacity within health systems [30]. During the foundational work phase (2018–2020), the facilitation team had one Lead, an academic highly regarded in the knowledge mobilization community. Subsequently, in 2020, AbSPORU adopted a new leadership model for this team, including two Co-Leads with additional leadership roles in AHS. These Co-Leads maintained their original roles in AHS while also taking on leadership roles in AbSPORU, creating a cross-sectoral dyad leadership model for the infrastructure, with leaders spanning academic and health services sectors.

## Methods

This study was designed to answer the research question, ‘how can facilitators coordinate existing implementation enablers to build implementation infrastructure in support of the evolving needs of LHS over time?’ To answer this question, we conducted a longitudinal case study of the Implementation Science Collaborative’s design and its first year of operations (2018–2021). Data were generated using document analysis and semi-structured interviews. Longitudinal data collection allows for a developmental perspective, in which researchers can monitor behaviours and contextual changes that impact the success of large-scale change initiatives [28, 31]. Longitudinal case study often incorporates mixed methods to give researchers the opportunity to provide descriptions of local contexts and variations in specific cases. This detailed description is crucial for understanding how and why certain activities support or hinder large-scale change [28, 32]. We followed the Standards for Reporting Qualitative Research (SRQR) [33] to guide these methods (Supplement 1).

### Data collection

#### Document analysis

We collected 190 infrastructure planning and operations documents (e.g., meeting minutes, annual action plans, internal reports) from October 2018 to December 2021. We reviewed and analyzed these documents to track infrastructure design decisions and strategies over time. A timeline of events (Supplement 2) was created from these documents to guide the triangulation and validation of our findings through semi-structured interviews.

#### Interviews

Between March and June 2022, we used the timeline to facilitate semi-structured interviews (*n* = 6) with Implementation Science Collaborative members who had been involved with the Implementation Science Collaborative from its conceptualization through its launch. Participants involved throughout the Implementation Science Collaborative co-design could provide the deepest insights into context and contextual changes that required response by the co-design facilitators. All potential participants who fit the inclusion (*n* = 6) were invited to participate and all agreed. Participants included Implementation Science Collaborative leaders (*n* = 2), AbSPORU staff involved in the co-design facilitation (*n* = 2) and Implementation Science Collaborative partners (*n* = 2). The interviews were conducted virtually over Zoom and ranged from 43–57 min in duration. The lead author conducted all interviews. In the interviews, we asked about a) participants’ roles and involvement with the Implementation Science Collaborative, b) their reactions to the events and results, c) specific co-design activities, d) co-design strategies employed, and e) future plans for the collaborative (Supplement 3). After each interview, we performed a member-checking exercise to ensure accurate understanding of responses. To enhance the rigour of our study, we summarized each conversation and returned it to the participants for verification [34]; all participants confirmed our understanding with no additional comments. By providing our own interpretation of the interviews, rather than providing the verbatim interviews for review, we capitalized on the expertise of participants to the fullest extent, as participants could comment on the findings that would be presented to wider audiences [34].

To further strengthen rigor, in September 2023 we held a final group interview to validate preliminary findings and ensure an accurate understanding [32] of AbSPORU’s management approach and the characteristics they used to facilitate the Implementation Science Collaborative co-design. Attendees included Implementation Science Collaborative leaders (*n* = 2), AbSPORU staff involved in co-design facilitation (*n* = 2), and an Implementation Science Collaborative partner (*n* = 1). Similar group interviews with service providers strengthen qualitative studies by providing additional insights into communicating with diverse parties in health contexts [35].

### Data analysis

Interviews were audio-recorded, transcribed verbatim, and reviewed for accuracy. Transcripts were then organized and coded using NVivo 11 analytic software [36]. We developed our a priori coding framework using Nystöm et al.’s adaptation of the Large-Scale Change Driver Model [28], originally developed by Perla et al. [27] to flesh out key constructs. This model is detailed in the following sub-section.

We used the Large-Scale Change Driver Model to deductively code the interviews. We also utilized inductive coding to account for data that did not fit the model [37, 38]. This created opportunities to flesh out the under-developed Large-Scale Change Driver Model constructs. The approach helped us uncover AbSPORU’s key strategies and the characteristics they leveraged to facilitate co-design of the implementation infrastructure. Moreover, the longitudinal approach, paired with this model, allowed us to observe the influences of leadership model changes described in the context section. With the coded interview data, we revisited the documents using a matrix that chronologically categorized planning activities as well as the different strategies and characteristics used to facilitate co-design. This allowed us to identify which strategies and characteristics were employed at specific points in time during the co-design process.

All study participants provided informed consent to be included in this research. The research design was approved by the University of Alberta Research Information Services, Research Ethics Board—Health Panel (ID: Pro00084611).

#### Theoretical framework

**Fig. 1:**
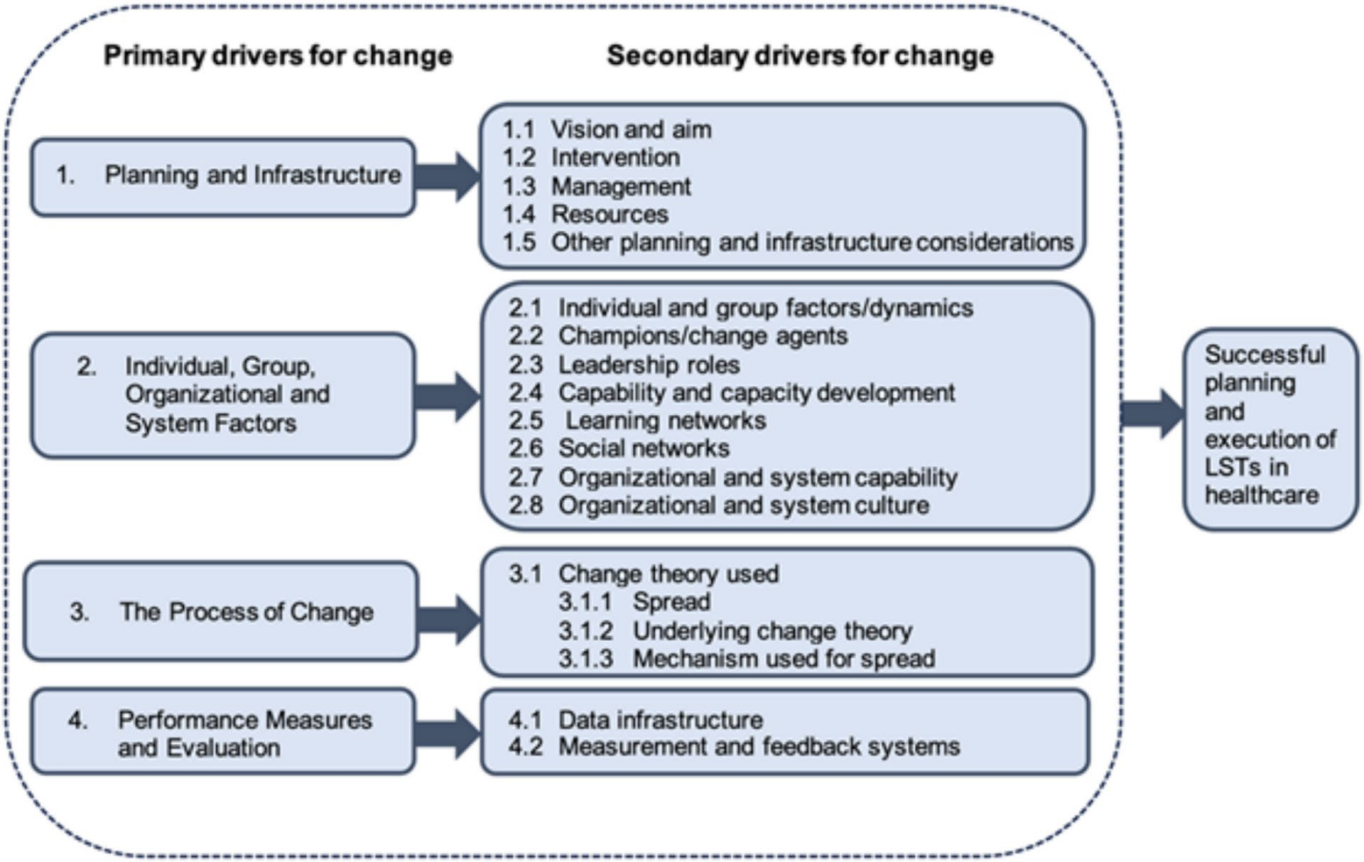
Large-Scale Transformation Driver Model Nyström et al.’s 2014 adaptation [28] of Perla et al. (2013) [34]

The Large-Scale Change Driver Model (Fig. 1) is an evidence-based model designed to support healthcare organizations in planning and implementing change within healthcare contexts [27]. The model includes four primary and sixteen secondary drivers relevant to large-scale improvement initiatives such as the Implementation Science Collaborative [28]. The primary drivers are 1) planning and infrastructure, 2) individual, group, organizational and system factors, 3) the process of change, and 4) performance measures and evaluation. These drivers are not mutually exclusive but serve as an inventory of considerations for teams to enact change.

The planning and infrastructure driver involves having a clear vision and goals, the intervention itself, strong management, sufficient resources to implement change, and additional planning and infrastructure considerations [27]. The individual, group, organizational, and system factors that drive the process address the human and relational dynamics that affect an organization’s capacity for change. This includes governance structures and processes to manage and sustain change (e.g., a functional implementation unit). The process of change driver outlines implementation principles, including the theory of change and mechanisms to create and spread desired changes. Lastly, the performance measures and evaluation drivers underscore the need for data and feedback systems to monitor and adapt to the implementation’s impacts over time.

This study focused on the planning and infrastructure driver, which, while reflecting normative recommendations from change and management literature [28, 39], lacks practical, evidence-based guidance for design and management strategies. Numerous teams have noted these gaps and called for more specifics on managing large-scale change [27–29]. By applying the Large-Scale Change Driver Model to the co-design process described in this study, we identified characteristics and strategies to establish strong management and supportive infrastructure, as the original model advocates.

## Results

Our analysis showed the infrastructure design and launch followed established principles of implementation planning and execution. Implementation intermediaries demonstrated their effectiveness as facilitators by using their expertise to guide co-design and implementation planning. They were also politically agnostic towards the resulting infrastructure, allowing them to mitigate power imbalances among the cross-sectoral actors involved in the co-design activities. However, intermediaries could not replace strong leadership. Cross-sectoral leadership accelerated and enhanced the partnership building required to conduct this work supporting the local learning health system.

### Infrastructure design activities

**Fig. 2:**
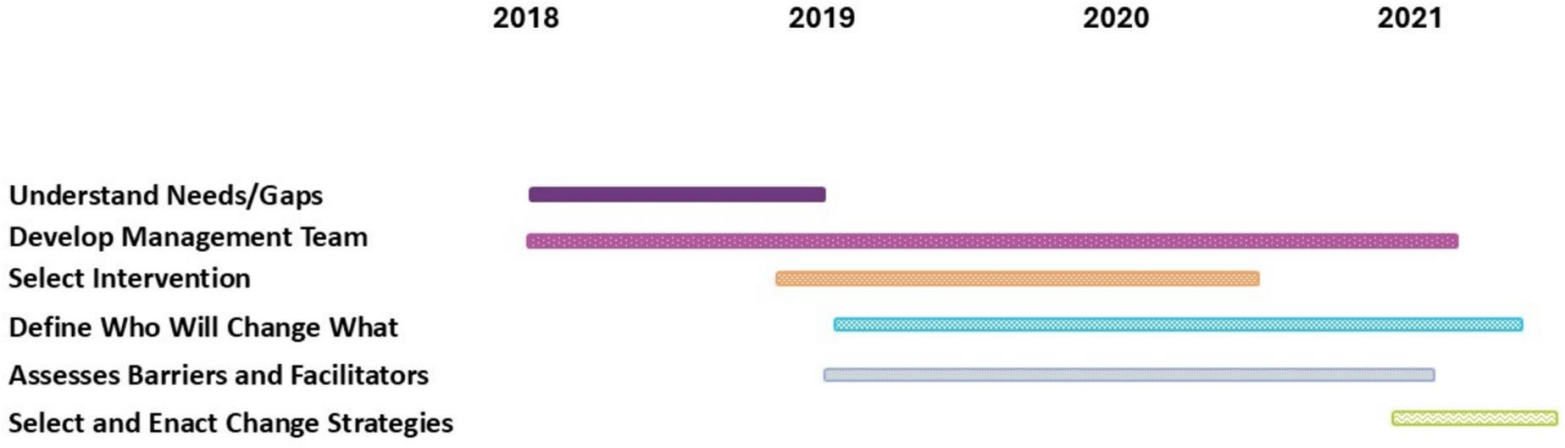
Design Process by Implementation Planning Procedural Steps

We used the documents to develop a timeline of activities that underpinned the infrastructure design and operationalization. The timeline illustrated a co-design process that aligned with implementation science planning principles, including exploring the initiative, identifying key partners, and assessing the initiative’s fit to context [7]. This process corresponded with the Large-Scale Change Driver Model’s planning and infrastructure construct. During this phase, the facilitators drew out partner insights about available resources, existing local implementation expertise, and complementary programs to be leveraged in developing the new infrastructure (Fig. 2).

Interview participants noted these co-design discussions as important milestones for articulating the vision of the infrastructure, what elements of implementation it would support, the various needs of the partners, and the context surrounding the forthcoming infrastructure.

“I think that that planning that multistakeholder planning committee that I sat on was helpful. An exercise of bringing all the different players together and building out a bit of a co-design about what this is going to look like for Alberta.” (Co-Design Partner 1)

“The co-design piece, I think is more around our internal group trying to figure out all the pieces…having conversations with all sorts of people who live in this space. How might you think about it? I think there was a fair amount of co-design in that space.” (Co-Lead 1)

The intermediaries managed all administrative aspects of the co-design (e.g., planning and facilitating meetings and developing meeting summaries and action plans). This created space for co-design partners to focus their conversations and contribute insights from diverse perspectives. The facilitators initiated efforts to help partners articulate the vision and objectives of the initiative and address power imbalances among partners. For instance, documents revealed that AbSPORU facilitators gathered insights to draft and revise a concept document outlining the infrastructure’s vision, operations, and target beneficiaries.

“It was a really good discussion, bringing all of those perspectives, because I always felt like the academics, all they really wanted to do was study implementation. And in AHS we have a real practical need to know the best way.” (Co-Design Partner 2)

“The meetings were quite interesting, because everybody who came to that room had a very different understanding of what this was. In fact, I don't think any of us really understood what [the idea for the infrastructure] was. I would say that one of the things that was quite successful was to learn from people who really understand what this is all about and what the end game is.” (Co-Lead 2)

“It was fantastic to be able to bring all of those different stakeholders together and it really is fascinating to listen to all of those different perspectives.” (Facilitator 1).

The facilitators also invested time in conducting foundational research to inform the infrastructure. They conducted informal interviews with healthcare leaders in other settings to learn about infrastructure models and key considerations. Additionally, they conducted a social network analysis to identify existing implementation research and practice partnerships across the province. For context, this work was led by the first author of this study and is currently under review. (Brooks et al., under review).

“That's how we would typically do things, but I think it was the right approach to map out the stakeholders in all the different communities and have that thinking in the approach.” (Facilitator 1).

“The asset mapping and the social network analysis was a huge undertaking… I would say a turning point really was after the asset mapping, which I think was a good way to give people something tangible. I think that was a really good thing to do.” (Co-Design Partner 2)

### Intermediaries as infrastructure facilitators

AbSPORU provided a core team of 2 to 3 intermediaries who acted as the central planners and facilitators for all co-design events and administrative activities involved with the infrastructure. Up to 10 additional AbSPORU staff assisted for different meetings and events. Throughout the co-design, the AbSPORU facilitators were accountable to AbSPORU and its broad mandate to respond to the health system in whatever ways the system requested. Once the Co-Lead model was introduced, facilitators reported to Co-Leads, who had additional insights into how to best serve the health system. Yet the facilitators maintained their broad mandate from their funder.

In their role as co-design facilitators, AbSPORU exhibited specific characteristics (Table 1). These characteristics allowed them to manage nearly all primary and secondary drivers of the Large-Scale Change Driver Model. This evidence demonstrates the substantial influence of co-design facilitation on infrastructure development. The facilitators committed to listening to diverse perspectives from their co-design partners and adapted their facilitation styles, communication, and models based on ongoing feedback, demonstrating their flexibility, nimbleness, and responsiveness to the health system.

**Table 1:** Facilitator characteristics throughout infrastructure design and launch

Document Source (*n*)	Design Stage	Characteristic	Large Scale Change Driver Model Constructs Touched	Illustrative Quote
42	Needs assessment	Value in alignment/ relevance	• Group Dynamics • Social Networks • Leader Actions • Intervention • Capability and Capacity Development • Champions and Change Agents • Leader Actions • Leader Characteristics • Opinion Leaders	“Definitely our first engagement event was a huge milestone. And I think, for me, what I took away was, first of all, people were excited about this. Because they showed up and stayed.” (Facilitator 1) “Acute Care Excellence really grew out of the Ernst and Young review, which was mandated by [the provincial ministry of health]. And that was, as we all know, mandated by certain political slant of the current provincial, we, so whereas the way we built the ISC was more bottom up in the sense of, like, if the results of the AISA discussion had gone in a completely different direction, that's where the initiative would have gone.” (Facilitator 2)
15	Select Intervention	Curiosity/ openness	• Group Dynamics • Intervention • Leader Actions	“So, I think there's still a bit of what I would call polarity between some of our members and others around why did you pick this? Or why are we focusing on that? And oh, by the way, that may work in your world AHS world, but it would never work in our primary care world. And I think that polarity is great.” (Co-Lead 1)
75	Needs assessment, selecting intervention, developing a management team, assessing barriers and facilitators, enacting change strategies	Responsiveness	• Intervention • Vision and Aims • Underlying Change Theory • Group Dynamics • Individual • Capability and Capacity Development • Social Networks • Organizational and System Capability	“The co-design piece, I think is more around our internal group trying to figure out all the pieces, like the Scientific Advisory Board, and having conversations with [our partners] who live in this space, how they might think about it. I think there was a fair amount of co-design in that space.” (Co-Lead 1) “I think it was very bottom up in the sense of we listened really attentively to what the leaders said as well we brought together people from a wide range of perspectives across the system, like within AHS within primary care, and we were guided by smart people who knew the politics much better than I ever did at that point.” (Facilitator 1)
33	Developing management team	Commitment in service to the health system	• Leader Actions • Resources • Underlying Change Theory • Other Planning and Infrastructure • Capability and Capacity Development • Leader Characteristics • Champions or Change Agents • Opinion Leaders	“The second thing that I think emerged was I think people thought this would be an entity owned by somebody. And I guess somebody has to own it. But I think the other positive thing that emerged is this is this is taking advantage of Alberta's assets, and coming together in a common sort of governance and understanding about how we could make our system better. But that that was an evolution over a couple of years.” (Co-Lead 2) “Then too seeing [AbSPORU’s] various drafts for their ongoing funding and seeing how centrally the ISC was written in, was also very interesting”. (Facilitator 1)
13	Developing a management team, selecting an intervention, defining who will change what, assessing barriers and facilitators, enacting change strategies	Systems thinking	• Capability and Capacity Development • Champions and Change Agents	"So, I thought that was a good approach. And then this whole asset mapping and the network analysis, I also thought was a really cool idea… And I think what I liked about it is it really did highlight where are our strengths and capabilities here in Alberta, and where do we need to pay attention to gaps or potential blind spots to the work…we had to do this foundational work."(Co-Lead 1)

The nature of knowledge mobilization intermediary work made AbSPORU well-suited for facilitating the design of collaborative infrastructure. Throughout the initiative’s co-design and launch, AbSPORU allocated time for numerous staff members from their knowledge mobilization/implementation team to support administrative and co-design facilitation tasks. These staff members had backgrounds in knowledge synthesis, translational research, community-based research, business, implementation practice, and implementation research. Such backgrounds equipped them with the skills to engage partners, map communities, generate ideas, manage group dynamics, communicate in plain language, develop prototypes, and evaluate outcomes. These skills proved essential for delving into Alberta’s health system implementation context, collecting insights from initiative collaborators, and developing infrastructure models for feedback and refinement.

“The facilitators were very skilled and persistent. T I think using different approaches over time, people started to get a better understanding of what this could potentially do for us in Alberta.” (Co-Lead 1)

“And we've had to bring together a huge amount of both practical and academic knowledge, which has been time consuming and it's hard work.” (Facilitator 1)

Additionally, AbSPORU’s intermediaries were politically neutral in the resulting infrastructure, enabling them to address power imbalances among cross-sectoral parties involved in co-design activities. Twenty-eight percent of the document dataset related to managing group dynamics. The interviews confirmed that AbSPORU’s role as a general support for the health system afforded them the latitude to manage these dynamics effectively.

“[AbSPORU’s] role was really to host the party. We were unique in that our mandate was just to be helpful and responsive to system needs and the flexibility that was built into the mandate… this wasn't in our work plan at all. We had the flexibility to recognize this important thing we could do, and nobody said, ‘Well, hang on. We didn't approve that; you can't do it.’ This is one of the preconditions that made this possible.” (Facilitator 1)

### Cross-sectoral leadership in support of the learning health system

While the intermediaries facilitated the initiative, a notable acceleration occurred when a cross-sectoral dyad, representing academic and health system expertise, assumed leadership of the infrastructure development. The Co-Leads consisted of 1) a senior leader form the provincial health authority with extensive experience in and oversight of health system quality improvement, and 2) an academic leader with a strong research portfolio in implementation science and health system innovation, holding joint appointments within the university and health services systems. Both Co-Leads brought deep organizational knowledge, broad networks, and leadership credibility. The Co-Leads’ networks, and knowledge of organizational structures and politics between all the different organizations involved, enhanced the facilitators’ efforts. These insights strengthened the facilitators’ abilities to manage group dynamics, anticipate tensions, and develop prototypes that better addressed the needs of different partners.

"I think a facilitator has definitely been [the Co-Leads]… what they bring in terms of connections within the Health Authority, Alberta Health Services, but also the awareness of how Alberta Health Services fits within the overall health system in Alberta… They knew what the different groups’ questions would be, or what their perspectives would be. And they were of course, well connected, very perceptive and insightful about implementation. They were able to give a lot of guidance." (Facilitator 1)

The Co-Leads also identified which potential partners held power within their organizations to endorse and support the infrastructure. Prior to their involvement, facilitators struggled to identify key players and establish contact. By developing and executing a plan to socialize the infrastructure concept to key partners, the Co-Leads secured endorsements and recruited key partners to form the initiative’s governance structure. These actions by the cross-sectoral leadership overcame the facilitators’ limitations and lent credibility to the overall infrastructure development.

“We always had some connections in the health system. But having [the Co-Leads] did open that up for sure. It was a facilitator having those kinds of critical people at the helm. They have a lot of credibility in the academic world and in the health system as well. Our primary facilitators were our existing relationships and the change in leadership.” (Facilitator 2)

Infrastructure co-design partners represented many organizations within the Alberta health system, each with distinct mandates and priorities. The co-design approach helped align these diverse directives. Despite challenges arising from differing opinions and preferred infrastructure designs, the facilitators and Co-Leads built relationships and understanding between the co-design partners.

*“*Yeah, well I think it maybe it's part of the co-design, but I think the inclusiveness of it has been, I think, a strategy that's been important to make sure that you're trying to get to all of the potential stakeholders*.”* (Co-Lead 2)

The Co-Leads were already involved with Alberta’s existing learning health system. Through their learning health system networks, they recruited key partners into the governance structure, facilitating organizational learning about implementation. For instance, they recruited leaders from embedded health innovation teams, quality improvement, data capture, and analytics. The Co-Leads also held positions that provided a comprehensive view of innovations that would benefit from implementation science insights. Their broad familiarity with the system helped other partners and facilitators understand the expertise needed to strengthen implementation across Alberta.

“We walked the academic and the operations. Finding members wasn't about tying up to operations alone. It was about having leadership who work on both sides and understand both sides.” (Co-Lead 1)

### Adaptation of the Large-Scale Transformation Driver Model

While the Large-Scale Transformation Driver Model proved useful for this study, using it to code an infrastructure and learning health system initiative revealed limitations to the model’s utility. Specifically, the model’s management constructs were too vague to develop sufficient insights into facilitating and managing change. Consequently, we refined the management construct by introducing three inductive codes that were more “fit for purpose”: management approach, management characteristics and management activities. These additions provided practical insights for facilitating large-scale change (Table 2).

**Table 2:** New management sub-codes and their definitions

Management Approach	The overall strategy or approach used to facilitate the intervention/infrastructure design and management
Management Characteristics	The qualities or features that enabled intervention/infrastructure facilitation
Management Activities	Specific events or activities that supported intervention/infrastructure design and management

Exploring the model through an implementation science perspective also clarified the definitions of the intervention construct. The original model conflated the terms intervention and implementation. Thus, we updated the code name to reflect the true nature of this activity: implementation planning (Supplement 4). Instead of focusing on which intervention was selected to create change, our updated codes shift the focus to, “*how* such interventions can be delivered reliably and consistently to all patients” [27] (p. 30).

## Discussion

Using the modified Large-Scale Transformation Driver Model, our results illustrated the sweeping nature of management in large-change initiatives in healthcare, such as introducing new implementation infrastructure. We define implementation infrastructure as a dynamic set of formal and informal arrangements—such as people, processes, tools and relational mechanisms—that support and embed implementation science capacity within complex systems [40–42]. In the Implementation Science Collaborative, this includes structured co-leadership, dedicated knowledge mobilization roles, formalized planning processes, and the integration of implementation science research within service delivery and learning cycles. Infrastructure in this view, is not merely a set of technical supports, but a socially embedded system [43] intentionally designed and sustained through organized, relational and adaptive work. Furthermore, our case study offered detailed insights into the co-design and management of implementation infrastructure initiatives. The evidence supports our argument that intermediaries are effective facilitators due to their training in co-design and implementation planning. Furthermore, the intermediaries can more easily mitigate power imbalances among cross-sectoral co-design partners because they are agnostic towards the resulting infrastructure. Nevertheless, effective implementation infrastructure, particularly in learning health systems, require cross-sectoral leadership to integrate partners and perspectives and to build organizational learning cycles.

Literature shows that implementation infrastructure can improve service delivery and research impact. For example, the U.S. Department of Veterans Affairs developed the Quality Enhancement Research Initiative, an implementation science laboratory to foster collaboration between researchers, healthcare leaders, and frontline providers [44]. This infrastructure facilitates continuous feedback loops, aligning research priorities with health system needs and accelerating the adoption of evidence-based interventions [44]. As a result, service delivery improved, and both patient outcomes and provider satisfaction increased through more relevant, practical interventions [44]. This example reinforces the emerging evidence that implementation infrastructure—when intentionally designed and relationally embedded—functions as a dynamic system that supports continuous learning, enables local adaptation, and sustains evidence-informed change across diverse contexts.

### Infrastructure Design Process

The results demonstrated that developing implementation infrastructure benefits from an implementation mindset. The AbSPORU’s knowledge mobilization team treated infrastructure as an intervention, applying structured co-design principles as a core system-level mechanism to facilitate alignment and adaptive planning. This approach necessitated multi-party conversations to articulate the purpose of the infrastructure, clarify who needed to be involved in design and implementation, and identify strategies to implement the new structure effectively. The Center for Implementation, a prominent implementation practice training center in North America, has coined this type of exploratory work as ‘Designing for Implementation' [2]. Structured co-design, particularly when relationally facilitated, functions as an implementation infrastructure mechanism by organizing the relational work of diverse actors, aligning priorities and values, and enabling system-wide capacity building [30]. As Metz et. al. argue, co-design is not merely an engagement strategy but a structured process that embeds implementation science principles early in infrastructure development, producing system-level effects such as increased readiness, role clarity, and shared accountability [30].

In academia, hybrid designs in implementation research aim to achieve similar results, where scientists and research partners collaborate on projects to gain better insights into how to successfully implement new interventions [39]. Designing interventions with future implementation in mind increases the likelihood of successful implementation and sustainability, reinforcing the theoretical premise that early-stage co-design strengthens implementation infrastructure and improves intervention outcomes [2, 41, 45]. This perspective also aligns with learning health systems scholarship, which advocates for embedding implementation research within healthcare systems to promote continuous learning and improvement [24, 46].

However, embedding implementation research poses challenges due to its collaborative nature, requiring involvement across academic-health services ecosystems [24]. Studies show that providing infrastructure is essential to increase clinician and practitioner engagement with implementation research as it enables people to understand what implementation research is, how they can participate, and the value that these efforts will bring to them, their teams, and their patients in the future [47].

This case study contributes to theory by illustrating that intermediary organizations mandated to strengthen local health systems, such as Canada’s SPOR SUPPORT Units, serve as vital facilitators for designing for implementation. This supports the theoretical understanding that intermediaries operationalize infrastructure by coordinating multi-stakeholder alignment and addressing power dynamics to sustain implementation capacity [30].

In our case, the Implementation Science Collaborative co-design process spanned approximately three years, from first conceptual conversations in January 2018 to the infrastructure launch in June 2021, reflecting the sustained facilitation and iteration required for complex system infrastructure. The time commitment included contributions from 2–4 AbSPORU staff members. During this period 1 of the staff dedicated 1.0 FTE to the co-design process and the others provided up to 0.4 FTE depending on the intensity of current activities, underscoring the considerable investment needed for foundational infrastructure design.

This timeline is consistent with guidance from the Active Implementation Framework, which notes that full implementation efforts often require 2–4 years and that the design phase itself may require up to two years depending on the intensity of engagement [41]. These findings reinforce the view that structured, relationally-facilitated co-design is a foundational system-level mechanism that drives the development and sustainability of implementation infrastructure.

### Intermediaries as Infrastructure Facilitators

AbSPORU committed their knowledge mobilization staff members’ time to facilitate the co-design of this initiative from the start. These staff members were well-suited due to their existing skills in co-design principles. A recent systematic review identified four key co-design aspects: 1) forming equal partnerships, 2) valuing all partners’ knowledge, 3) using creative approaches, and 4) employing interactive prototyping [48]. AbSPORU facilitators committed to these aspects by practicing attentiveness and responsiveness, with a careful eye to ensuring that the co-design experience was productive, psychologically safe, and valuable to the partners involved. These results suggest that other facilitators, despite having a background in knowledge translation background, could perform this role if they practiced simpler attentiveness and responsiveness. Such practices and characteristics align with the competencies and functions of implementation support practitioners.

In this case, intermediaries operationalized implementation infrastructure by facilitating multi-stakeholder engagement, managing power dynamics, and translating diverse partner perspectives into shared plans. Their neutral positioning within the system allowed them to align interests, reduce tension, and promote collaborative decision-making, ultimately shaping a shared infrastructure vision. These functions closely align with the concept of “bridging factors” which is a domain in the Exploration, Preparation, Implementation and Sustainment implementation science framework and refers to the structures, processes, and roles that connect outer system and inner organizational contexts to enable implementation [42, 49]. Intermediaries in our case acted as “bridging actors” who enacted these bridging factors in practice, helping align values, priorities, and strategies across system levels. This work also reflects the role of knowledge brokers, who support relational and technical exchange across organizational boundaries [50], and boundary spanners who promote collaboration and trust across divides [51]. Furthermore, intermediaries enacted trust brokerage by building and maintaining relational trust among actors with diverse interests and positional power. These findings build on Metz et al.’s theory that implementation infrastructure includes relational and adaptive components—not only technical ones—by illustrating how intermediaries structure and support these relational dynamics in practice [41].

One type of knowledge mobilization intermediaries are implementation support practitioners who specialize in assisting with implementing evidence-based interventions. To perform this work, implementation support practitioners identify and contextualize implementation strategies to improve the likelihood of successful implementation and sustainability [30]. These practitioners are guided by the principles of practicing empathy, curiosity, commitment, equity, critical thinking, and cross-disciplinary approaches [30]. Our results directly align with this description of principles to support change. Consequently, our results suggest that implementation support practitioners can be highly valuable in facilitating infrastructure development and implementation—especially when sustained over multi-year timelines that allow for iterative, relational co-design. In this way, implementation support practitioners act as “infrastructure-as-function,” performing dynamic organizing work that brings stakeholders together and facilitates systems alignment [30]. This function aligns with boundary spanning, as described by Long et al. [52], who highlight how boundary spanners navigate complex relational dynamics to build collaborative networks across divides.

Another important finding from this study was that intermediaries were well-suited to facilitate infrastructure design when they were agnostic to the resulting infrastructure. Many organizations employ full-time knowledge mobilization and implementation support practitioners to assist with change efforts [53]. Although evidence shows that embedded implementation supports improve implementation outcomes [54], our evidence illustrated the power of having intermediaries who were external yet well-connected to the system facilitate co-design, as these intermediaries effectively managed group power dynamics and interests while gleaning important insights into the functioning of the emergent implementation infrastructure.

Theoretically, our results suggest that intermediaries are especially effective in infrastructure design when they occupy in-between positions—neither fully embedded nor fully external—enabling them to broker trust and navigate competing interests without being constrained by organizational mandates. This “in-between” or boundary position allows intermediaries to perform institutional work, including managing power relations and legitimizing new structures [40]. Thus, implementation infrastructure is not only built but enacted over time through the sustained facilitative work of intermediaries who connect, align and adapt system components.

### Cross-Sectoral Leadership in Support of the Learning Health System

The facilitators brought an implementation science lens to infrastructure planning. The Co-Leads brought contextual insights and networks of organizational relationships critical for integrating the infrastructure into existing learning health system processes. Our evidence shows that the Co-Leads played a champion role, which lent credibility and, in turn, buy-in for innovation uptake [55]. This case study also highlighted the additional benefits of cross-sectoral leadership. Our findings indicated that cross-sectoral leadership was a key facilitator of implementation infrastructure spanning research and practice organizations. The Co-Leads in this study established direct ties between health systems and academia and provided critical insights into partnerships and learning health system activities.

The Co-Leads’ cross-sectoral leadership ensured AbSPORU had the knowledge and connections necessary for successful implementation. Traditional dyad models are often hailed in healthcare delivery literature for responsibility sharing, leveraging multiple areas of expertise, and managing conflicts [56]. The Co-Lead model described in this case study enabled these traditional capabilities and further strengthen the dyad by co-locating the leadership with both the local health delivery authority and AbSPORU. The Co-Leads understood the institutional boundaries and working styles of researchers and practitioners who could access this infrastructure. This leadership model ensured the Implementation Science Collaborative fit well within the larger learning health system context.

The Co-Leads planned implementation in a learning health system by taking stock of the people and organizational capabilities and identifying where the new infrastructure fit. This work allowed implementation research questions to be embedded into existing healthcare innovation initiatives and that lessons learned could be shared widely to strengthen implementation approaches across the health system. Establishing these processes and infrastructure is essential for effective learning health systems [57]. Furthermore, learning health systems literature highlights implementation science as a crucial component for organizational learning, managing change, and supporting feedback loops that adapt and improve health service delivery over time [58–60]. Literature also shows that promoting infrastructure to strengthen implementation is essential for organizations to engage in implementation research [61]. Thus, promoting infrastructure is essential for high-quality learning health systems. The Co-Leads’ wide networks ensured this awareness was possible.

Our findings support a socially grounded understanding of implementation, where infrastructure is not just a technical tool, but a product of organized relationships, trust, and alignment across systems. The Co-Lead dyad brought together people, knowledge, and processes across institutional lines, helping to bridge academic and health system worlds through shared accountability and relational credibility. Their embedded yet spanning position allowed them to coordinate efforts between actors who might not otherwise collaborate, shaping how the infrastructure was understood, supported and sustained.

Rather than functioning solely as champions, Co-Leads performed the role of “boundary spanners” [52], who traverse organizational domains, align incentives, and establish partnerships across sectors. This supports scholarship on bridging actors who help connect implementation efforts to broader system dynamics by translating across roles, expectations and institutional logics [42, 43]. Taken together, these findings highlight how implementation success is shaped by the organized activities of the wider system—where relationships, leadership, and legitimacy matter as much as structure. Thus, the Co-Lead model offers an example of how leadership arrangements can be intentionally structured to perform infrastructure alignment work in learning health systems.

### Significance, Strengths, and Limitations

While grounded in a single case, this study contributes to mid-range theory by identifying key components of implementation infrastructure development. We highlight three interrelated elements: 1) the roles of intermediaries positioning, 2) relational co-design facilitation, and 3) cross-sectoral leadership alignment. These components represent dynamic and interactive pathways through which implementation infrastructure emerges and operates within complex systems, warranting further testing and refinement across other learning health systems and policy contexts. Beyond illustrating the utility of intermediaries and cross-sectoral leadership in developing implementation science infrastructure, our study advances change theory by elaborating on the constructs of the Large-Scale Transformation Model, specifically in the areas of change management and leadership. Consequently, our work enhances the model’s utility for managing large-scale change aligned with the quintuple aim often guiding healthcare improvement efforts [18].

Our results confirmed the importance of taking a fit for purpose approach to managing implementation systems. By developing a nuanced characterization of management, including approaches, governance structures, characteristics, practices, and activities, the model now supports strategic management decision-making to guide co-design in large-scale change initiatives. Similarly, the present study clarified pertinent elements of leadership roles in large-scale change. Our results showed that characteristics, actions, networks, and position within the system all positively impacted infrastructure design.

Importantly, our findings highlight that implementation infrastructure consists not only of technical tools, but also of organized relational and adaptive social processes that function dynamically to embed implementation science capacity within complex systems. Infrastructure characterized by strong relational and adaptive work is more likely to be sustained and produce continuous learning. Members of the research and authorship team (SB, DT, SM, CA) were also part of the Implementation Science Collaborative management team. To mitigate our bias and ensure trustworthiness, we brought our in-depth knowledge of the Collaborative to our analysis and gave participants the opportunity to review and comment on our interpretation of the results at two separate timepoints.

Importantly, our study sheds light on how learning health systems bring valuable knowledge to this model. The original authors of the Large-Scale Transformation Model noted learning as a critical component in healthcare change initiatives [27]. Our examination adds to this understanding by demonstrating how cross-sectoral leadership establishes learning communities that can inform and strengthen organizational change efforts through implementation science.

## Conclusions

The study findings underscore the practicality of a co-design approach facilitated by intermediaries to support local implementation of science infrastructure development. This approach helps to explore and find infrastructure designs that address diverse user needs. However, co-design is a complex process that benefits from intermediaries’ skills in brokering relationships, exploring context, and planning implementation. Furthermore, newly introduced infrastructures benefit from cross-sectoral leadership who can position and promote implementation science infrastructure across research and practice partners. In terms of the future we hope to continue to explore how these management systems work in practice as well as understanding the importance of context for scaling implementation infrastructure across different settings and populations. Linking implementation science as a key pathway for realizing better health outcomes and ultimately impact for our communities is an important feature of future work.

## Supplementary Information

Supplementary Material 1.

Supplementary Material 2.

Supplementary Material 3.

Supplementary Material 4.

## Data Availability

To protect the confidentiality of the participants study data are not publicly available. Anonymized data are available from the corresponding author upon reasonable request.

## Abbreviations

AHS: Alberta Health Systems
AbSPORU: Alberta Strategy for Patient Oriented Research SUPPORT Unit
