# Supplementary material for: Leveraging intermediaries’ skillsets to build implementation research and practice infrastructure: a qualitative case study
Source: Implement Sci Commun. 2025 Aug 2;6:80. doi: 10.1186/s43058-025-00765-2 (PMC12318380; doi:10.1186/s43058-025-00765-2)
Supplement: Supplementary file 2 — Supplementary Material 2. [file 43058_2025_765_MOESM2_ESM.docx]

| **Implementation Infrastructure Timeline of Main Events** | |
| --- | --- |
| **PHASE 1:** | **Exploring the possibilities and potential for a new implementation research infrastructure in Alberta** |
| Oct 2018 | *Advancing Implementation Science in Alberta Event #1 (75 attendees)*  Two day stakeholder engagement event showcasing implementation science work in Alberta and exploring the feasibility, scope, and support required to establish a partnership-based implementation research infrastructure in Alberta.   - **Purpose**: This event served as a readiness assessment for new implementation research infrastructure in Alberta. - **Outcomes**: SOAR (Strengths, Opportunities, Aspirations, Results) analysis completed and minimum specifications for an implementation research infrastructure Alberta were established. Alberta Identified as an enabling implementation science research context. |
| Nov 2018 - April 2019 | *Struck a multi-stakeholder planning committee for Advancing Implementation Science in Alberta 2.0*  AbSPORU, Strategic Clinical Networks, Alberta Innovates, and University of Alberta representatives searched literature for guidance on establishing implementation science labs (very little found), looked for similar guidance in learning health systems literature (again no practical guidance). To identify steps to establish an implementation science lab, the planning committee decided to use Advancing Implementation Science in Alberta 2.0 to develop a value proposition for an implementation science lab in Alberta.   - **Purpose**: Maintain momentum built at the first Advancing Implementation Science in Alberta event in building an Alberta-based implementation lab. - **Outcomes**: Planned event to bring key stakeholders together to discuss specific needs to be met by an implementation science lab and logistical considerations for establishing the lab. |
| April 2019 | *Advancing Implementation Science in Alberta Event #2* (50 attendees)  Facilitated stakeholder engagement with members from academia, Strategic Clinical Networks, Primary Care Networks, healthcare operations, Alberta Innovates, Alberta Health Services, and health research institutes. Event included a value proposition activity and discussions of logistical and research needs required to establish an implementation science lab in Alberta.   - **Purpose**: Decide whether or not to go ahead with formal implementation science infrastructure co-design in Alberta. - **Outcomes**: Multi-stakeholder attendees gave support for implementation infrastructure development. Conditions of moving forward included: 1) create infrastructure using collaborative/partnership-based approach; 2) organize the implementation research infrastructure activities in ways that did not require extra funds or a new office (i.e. avoid new silos); 3) do due diligence by conducting foundational research to establish the infrastructure. Alberta SPOR SUPPORT Unit took ownership as infrastructure design facilitation and host. |
| Oct 2019 - Nov 2020 | *AbSPORU writes Implementation Research Infrastructure into SPOR Phase II Application*  AbSPORU commits time and funding to infrastructure development as the priority initiative of SPOR Phase II.   - **Purpose**: Build implementation research infrastructure to support Alberta’s learning health system. - **Outcome**: AbSPORU able to provide highly qualified personnel to the facilitation and operations of the implementation research infrastructure. |
| Nov 2019 | *Conduct informal interviews with other implementation labs*  Spoke with health system partners about benefits and logistics of partnering with implementation science labs.   - **Purpose**: Collect key information on facilitators and barriers to establishing and maintaining implementation research partnerships and work. - **Outcome**: Interviewed members from Health Quality Ontario and Translating Research in a dental setting Initiaive. Learned how to plan for the cultural and financial issues that need to be smoothed out to perform implementation research infrastructure functions. |
| **PHASE 2:** | **Co-design Implementation Research Infrastructure Operations** |
| July - Nov 2019 | *Early Governance Structure Discussions*  4 meetings held between AbSPORU, local health innovation funders, Strategic Clinical Networks, academic implementation scientists   - **Purpose**: AbSPORU discussed integrating AbSPORU, academics, and Strategic Clinical Networks in a governance model that had a academic-health system dyad leadership. - **Outcome**: AbSPORU began working on an infrastructure Concept Document to clarify functions of the infrastructure and roles of the actors working in the initiative. |
| Nov 2019 | *Recruitment for 2020 Canadian Institutes of Health Research Health System Impact Fellow begins*  AbSPORU prepared postings for an implementation scientist and knowledge synthesis researcher.   - **Purpose**: Recruit people to build a business plan for the implementation research infrastructure - **Outcome**: No candidate found for either position. AbSPORU assigned as facilitator for all subsequent implementation research infrastructure planning activities. |
| Nov 2019 - Dec 2020 | *Asset Mapping and Social Network Analysis of Implementation Science and Support Communities*  AbSPORU staff cataloged all known implementation support practitioners and scientists working in Alberta. They also surveyed these contacts to learn who else was in the community and who actively collaborates with whom to complete implementation support and science in the province. A social network analysis was performed on the survey data to visualize the implementation community.   - **Purpose**: Understand capacity strengths and gaps in the province to inform how the implementation research infrastructure would plan and conduct implementation science. - **Outcomes**: The survey data gave us a baseline of perceived implementation science uptake in the province and an asset inventory of implementation support practitioners and scientists. AbSPORU learned that there are distinct differences in the ways that implementation support and science communities work together, warranting follow up interviews with survey participants to learn what facilitates or hinders collaborative implementation science in Alberta (interviews planned for Spring 2021). |
| February 2020 | *Day-Long Meeting Between AbSPORU, Strategic Clinical Networks, and Academic Partners*  AbSPORU met to discuss the AbSPORU Phase II application and implications stemming from the Ernst & Young report recommendations.   - **Purpose**: Establish 2020-2022 remit for implementation research infrastructure - **Outcomes**: Formed the core membership of the implementation research infrastructure Steering Committee. Set a plan to flesh out the Steering Committee, recruit members to the Scientific Advisory Board, finish the Concept Document, socialize the implementation research infrastructure to key stakeholder groups, complete implementation science prioritization, and plan an Advancing Implementation Science in Alberta 3.0 event. |
| Sept 2020 | *Health system partners become leadership dyad for AbSPORU LHS Team*   - **Purpose & Outcome**: Create formal link between AbSPORU and Alberta Health Services to grease relationships between Strategic Clinical Networks and AbSPORU Implementation Science & Data expertise. |
| June - Sept 2020 | *Implementation Research Infrastructure Model and Associated Concept Document Developed*  The Steering Committee and a writing sub-committee worked to develop and articulate the structure, functions, directions, and processes (e.g. project identification) of the implementation research infrastructure.   - **Purpose**: Create a succinct reference document to guide implementation research infrastructure processes and socialize the implementation research infrastructure within the broader health research ecosystem in Alberta. - **Outcome**: AbSPORU co-developed a clear working model to test and refine. The model included functions, roles, and approaches (e.g. integrating implementation science and quality improvement) of the implementation research infrastructure. |
| June - Dec 2020 | *Implementation Research Infrastructure Steering Committee Membership Fleshed Out*  The Steering Committee identified missing stakeholder representation and recruited additional members to fill those gaps on the Steering Committee.   - **Purpose**: Ensure that key stakeholders were at the table to co-design a feasible and useful implementation research infrastructure initiative. - **Outcomes**: The Implementation Research Infrastructure Steering Committee now has representation from AbSPORU, Strategic Clinical Network leadership, primary care, public health, indigenous wellness, Alberta Health Services quality improvement, ConnectCare and analytics, patients, Alberta Health Services operations, University of Alberta, University of Calgary and Alberta Health. |
| Sept - Nov 2020 | *Socialized the Implementation Research Infrastructure to Key Stakeholders in AHS*  Used the concept document and individual engagement meetings to discuss the implementation research infrastructure with decision-makers.   - **Purpose**: Socialize the implementation research infrastructure to decision-makers in Alberta Health Services implementation at the provincial level (Quality and Safety Outcomes Committee, e-Simulation, CancerControl, Improving Health Outcomes Together [iHOT] Team) and zone level (Integrated Quality Management) - **Outcomes**: Gained endorsement for the implementation research infrastructure concept, built relationships with key decision-makers, and collected feedback on further refinement of implementation research infrastructure functions and processes. |
| Nov 2020 | *Recruitment for 2021 Canadian Institutes of Health Research Health System Impact Fellow begins*  Learning Health Systems Team prepares postings for an implementation scientist and knowledge synthesis researcher   - **Purpose**: Recruit people to complete implementation research infrastructure support work (e.g., provide implementation science expertise to implementation teams, do knowledge synthesis for implementation research infrastructure generally or specific innovation projects). - **Outcome**: AbSPORU implementation scientist identified to support implementation research infrastructure. Implementation scientists writes an Health System Impact Fellowship Application application with AbSPORU and University of Alberta. No candidate found for Knowledge Synthesis position. |
| Nov 2020 - Feb 2021 | *Established the Implementation Research Infrastructure Scientific Advisory Board*  The Steering Committee used a list of key implementation science and learning health system competencies to identify potential board members and reached out to those in our existing networks.   - **Purpose**: Compose a Board with a range of backgrounds and skills that could help support cross-sectoral implementation science research. - **Outcomes**: AbSPORU recruited members with expertise in data analytics, normalization process theory, de-implementation, health technology assessment, indigenous research, long term care, primary care, audit & feedback, adaptation, and chronic disease. |
| Jan - Apr 2021 | Implementation Research Infrastructure Steering Committee Refines the Implementation Research Infrastructure Model  The Steering Committee refines the implementation research infrastructure model based on feedback from Fall 2020 engagement.   - **Purpose**: Clarify project identification process and roles of the Working Group and Scientific Advisory Board. - **Outcomes:**    - Refined implementation research infrastructure remit (e.g., implementation research infrastructure works to integrate implementation science and quality improvement, studying data efficiency outside the scope of implementation research infrastructure but will be pursued by AbSPORU).   - Steering Committee to create high level priorities instead of specific implementation science questions.   - Working Group to find and propose projects for implementation research infrastructure support.   - Scientific Advisory Board given a spectrum of participation opportunities based on interest (from high level strategic direction to embedded research opportunities). |
| May 2021 | Implementation Research Infrastructure Working Group Established  Includes members from AbSPORU, Strategic Clinical Networks, Health System Impact Fellowship, Alberta Health Services |
| June 2021 | *Implementation Research Infrastructure Launch Event*  Two-part event starting with a Scientific Advisory Board orientation of the implementation research infrastructure followed by an all-members meeting to discuss a high-level work plan for the rest of 2021.   - **Purpose**:  1. Orient the Scientific Advisory Board to the implementation research infrastructure model. 2. Familiarize Implementation Research Infrastructure, Steering Committee, Scientific Advisory Board, and Working Group with one another and with AbSPORU leads. 3. Develop June-December 2021 implementation research infrastructure work plan.  - **Outcomes**: Identified key activities to be completed prior to project identification (i.e., prioritization principles and key implementation issues list). |
| June to Nov 2021 | *Foundational Work Completed for Implementation Research Infrastructure Operations*  The Steering Committee, Scientific Advisory Board, and Working Group work together to develop prioritization principles and list of priority implementation science issues.   - **Purpose**: The prioritization principles were developed as an implementation research infrastructure transparency mechanism. The priority implementation issues were developed as high-level guidance for the Working Group to identify potential projects for implementation research infrastructure support. - **Outcome**: The principles and the issues list were endorsed by all implementation research infrastructure groups |
| July 2021 - current | *Health System Impact Fellowship Position Awarded - Starts Working with the Learning Health System Team*  *AbSPORU puts together Health System Impact Fellowship application with the University of Alberta to study implementation of Acute Care Bundles*   - ***Purpose****: The fellow was to bring implementation science capacity into the province, sit on the* implementation research infrastructure *working group, and support* implementation research infrastructure  *priorities.* - ***Outcome****: Supported the 2021-22* implementation research infrastructure *priorities around impact of co-design in the Acute Care Bundles initiative.* |
| Dec 2021 | *All Members Meeting*  All-members meeting to shift into implementation research infrastructure operations.   - **Purpose**: To test the applicability of the co-designed implementation research infrastructure operations tools (i.e., the prioritization principles, issues list, and project identification process). - **Outcomes**: Testing the tools using real-world Alberta Health Services initiatives highlighted that there needs to be flexibility to support projects at different stages of development and with different degrees of implementation science capability. |
| Dec 2021 | *Working Group Assigned Acute Care Bundles to Support*  The Working Group continued to struggle to identify the best avenues and processes for project identification. The Learning Health System Team worked to facilitate further discussions with Strategic Clinical Networks Assistant Scientific Directors and Scientific Directors to help identify exactly how the Working Group could perform its project identification tasks.   - **Purpose**: Working Group assigned to support the Acute Care Bundle initiative as a demo project to identify and sort through operations sticking points. - **Outcomes**: Sub-Working Group struck to support the quantitative measurement of co-design outcomes of the initiative. Operations development ongoing into 2022. |
