# Supplementary material for: Leveraging intermediaries’ skillsets to build implementation research and practice infrastructure: a qualitative case study
Source: Implement Sci Commun. 2025 Aug 2;6:80. doi: 10.1186/s43058-025-00765-2 (PMC12318380; doi:10.1186/s43058-025-00765-2)
Supplement: Supplementary file 3 — Supplementary Material 3. [file 43058_2025_765_MOESM3_ESM.docx]

Interview Questions – IS Collaborative participants and members

Thank you very much for agreeing to participate in this study. AbSPORU appreciates you taking the time to share your experiences in helping to establish Alberta's Implementation Collaborative. Your experiences using our services and/or in the broader knowledge translation and implementation science fields are extremely valuable. Please feel free to share any and all ideas you have on how we can continue to support KT and IS in Alberta as they come to mind throughout this interview.

**Part 1: Initiative Background**

1. **Can you describe your role with AISA and the IS Collaborative? Please be specific about the nature and level of your involvement.**

[PROBING QUESTIONS]

1. When did you start working with the initiative? Why did you agree to come onboard?

2. Has the level or nature of your involvement changed at all since you started? If so, Please describe how and why.

1. **Tell me about your experience being part of AISA and the IS Collaborative during the early AISA meetings and the co-design of the ISC.**

[PROBING QUESTIONS]

1. How has this experience been different than other co-design work you have been a part of?

2. For you, what processes or activities did you liked or what went well?

3. What did not go so well? Have these problems been corrected or are they ongoing?

**Part 2: Reactions to Events and Results So Far**

**4. In your opinion, what were the key milestones for the ISC in terms of co-design?**

[PROBING QUESTIONS]

1. Why were these specific events important?

2. What were their significance for moving forward?

**5. Can you describe what you think facilitated the co-design process?**

[PROBING QUESTIONS]

1. What conditions existed to facilitate successful co-design?

**6. What were the challenges to co-designing the ISC?**

[PROBIN QUESTIONS]

1. How have you and the rest of the team addressed these challenges?

2. Hindsight being 20/20 would you have done anything differently if you could go back and try again?

**Part 3: Interventions and Activities**

I would like to talk about some of the specific ISC co-design activities that took place.

**7. Here is a list of events and meetings that took place leading up to the formal launch of the ISC as well as activities that took place between them. What do you think were the critical pieces that required co-design?**

[PROBING QUESTIONS]

1. In which of these activities did we do co-design well? What indicates that we did co-design well here?

2. Where did we fail to do co-design well? What was the outcome of this failure?

- Who else was involved in each of these activities?
- Was anyone missing? If so, who and what do you think would have been different if they were involved?
- What was the goal of the activity? Did it work out as anticipated? Why/why not?

**8. What is missing from this list that had to happen to get the initiative to where it is today?**

[PROBING QUESTIONS]

1. What partnerships had to be developed?

2. What decisions had to be made?

3. What infrastructure was built?

4. Was there any training involved?

5. Other required activities? Collaborations, meetings, support-building, hiring, events, presentations, communication strategies designed, environmental scans, team building, outreach, other research?) *generate a living list that activities can be added to as participants bring them up*

**Part 4: Strategies**

**9. Can you identify and describe any strategies in addition to co-design that have been developed or employed to support or facilitate the overall efforts of the IS Collaborative so far? (e.g. careful use of language, clearly distinguishing clinical research from implementation research, following health system priorities, recruiting SAB members we already knew, etc.)**

[PROBING QUESTIONS]

1. Can you give me an example or two of the strategies that really worked well? Why do you think these were so successful?

2. Were any strategies tried but were ultimately unsuccessful? What do you think were the barriers to these working as hoped?

**Part 5: Future Plans**

**11. What are the next steps required to keep pushing the IS Collaborative forward?**

[PROBING QUESTIONS]

1. Who and what is currently involved in these next steps?
2. Is anyone missing? Do you have a sense of what barriers are keeping them from participating? If so, what are they?
3. Are there any seemingly insurmountable barriers right now? Do you know if there are plans to address these? If so, what are they?
4. In your opinion, what are some of the key facilitators or opportunities for the next steps of the IS Collaborative?

**Final Questions**

**12. Is there anything else you would like to add about your experience working in this initiative or trying to embed implementation research into the health care system in Alberta?**
