# Supplementary material for: Leveraging intermediaries’ skillsets to build implementation research and practice infrastructure: a qualitative case study
Source: Implement Sci Commun. 2025 Aug 2;6:80. doi: 10.1186/s43058-025-00765-2 (PMC12318380; doi:10.1186/s43058-025-00765-2)
Supplement: Supplementary file 4 — Supplementary Material 4. [file 43058_2025_765_MOESM4_ESM.docx]

Adapted Codebook Developed from the Large-Scale Change Driver Model from Perla et. al. Adaptations in **Bold**

| Code | | | Definition |
| --- | --- | --- | --- |
| Planning and Infrastructure | Vision and aims | | Large-scale change initiatives appeal to constituents through the use of compelling visions and aims. Organizers should turn high-level strategy into specific goals with real deadlines. |
|  | Intervention - **Implementation** | | **Explicit guidance on *how* to change and not just *what* needs to change. This includes the elements of Everett Rogers’ diffusion of model: relative advantage, compatibility, simplicity of use, trialability, observability of the benefits and reinvention. Implementation or the strategies used to operationalize the large-scale change initiative Excluded guidance on what needs to change and instead included this in the underlying change theory code.** |
|  | Management | Management | Elements of overall management of large-scale change (e.g., managing system levers, policy changes, measurements systems, and responding to policy changes). |
|  |  | **Management Approach** | **The overall strategy or approach used by organizers to facilitate the design of the large-scale change initiative.** |
|  |  | **Management Characteristics** | **The qualities or features that enabled the organizers to successfully build and facilitate the large-scale change initiative.** |
|  |  | **Management Activities** | **Specific events or activities that the organizers undertook to facilitate the design of the large-scale change initiative.** |
|  | Resources | | Resources required by both the adopters/users and organizers to make the change. This includes: personnel, project management, time, funding, and investment in infrastructure and training. Reduction of resource needs by the initiative partners/users fits here. |
|  | Other planning and infrastructure | | Information technology (IT), environmental factors, such as community socioeconomic infrastructure. |
| Individual, Group, Organizational and System Factors | Individual and Group Factors or Dynamics | **Individual Factors** | How individuals engage with the innovation and affect adoption of change. Includes, personal values, workload increases/decreases, willingness to change, ability to use innovation, etc. |
|  |  | **Group Factors** | Implementation capacity, decision-making authority, leadership, good timing for change, engagement (a highly credible evidence base; a genuine belief that the new model provides better patient care; and an appetite for the innovation). |
|  | Champion and Change Agents | **Champion/Change Agents** | People who model new behavior. |
|  |  | **Opinion Leaders** | People who influence thinking about the large-scale change initiative. |
|  | Leadership Roles | **Leadership Characteristics** | **Characteristics of strong leadership (e.g., charisma, positive attitude, senior policy and delivery support, consistent vision across leaders, persistence, commitment, continuity, style and collaboration.)** |
|  |  | **Leadership Actions** | **Actions of strong leaders that facilitated/inspired change uptake. This includes engaging staff, articulating the vision to the workforce, identifying the target population, making the work a priority, committing time and resources to achieve objectives, aligning organizational goals, etc.** |
|  | Capability and capacity development (Individuals) | | Individual skills (e.g., training) to enable the workforce to improve healthcare quality and productivity at scale. |
|  | Learning networks | | Learning networks that maximize workforce improvement capability. E.g., formal training such as structured learning environments. |
|  | Social networks | | Social networks to generate motivation and increase energy for improvement and aid knowledge exchange. E.g., informal knowledge sharing opportunities such as communities of practice. |
|  | Organizational and system capability | | Organizational ability to plan and deliver widespread innovations. Scale and spread capabilities. |
|  | Organizational and System Culture | | Positive cultural characteristics (e.g., enablement of cross functional teamwork). Cultural behaviours that contribute to a culture of adoption and spread (e.g., KT). |
| The Process of Change | Change Theory Used | Spread | The extent to which the effort is actively pushed to participants. |
|  |  | **Underlying Change Theory** | The underlying model that drives the work of the large-scale change initiative. **The theoretical framework that guides how the large-scale change initiative is supported, i.e., *what* needs to change.** |
|  |  | Mechanism used for spread | The mechanism used to spread the intervention. |
| Performance Measures and Evaluation | Data infrastructure | | Data processes and infrastructure in place to collect reliable and valid data, and link change initiatives to results. |
|  | Measurement and feedback systems | | Methods and capability to design and conduct evaluation of change and to share results back. |
